# Bacterial Isolates and Characteristics of Children With Febrile Neutropenia on Treatment for Cancer at a Tertiary Hospital in Western Kenya

**DOI:** 10.1200/GO.23.00313

**Published:** 2024-02-01

**Authors:** Samuel Kipkemoi Kipchumba, Festus Muigai Njuguna, Winstone Mokaya Nyandiko

**Affiliations:** ^1^ Department of Child Health and Paediatrics, Moi University College of Health Sciences, Eldoret, Kenya; ^2^ Academic Model Providing Access to Healthcare, Eldoret, Kenya

## Abstract

**PURPOSE:**

This study aimed to identify the patient characteristics of children with febrile neutropenia, the associated bacterial organisms, and their sensitivity patterns.

**MATERIALS AND METHODS:**

A descriptive cross-sectional study was conducted at the Moi Teaching and Referral Hospital (MTRH) pediatric oncology ward, from June 2021 to April 2022. A total of 110 children who developed fever and neutropenia during chemotherapy were enrolled. Blood samples for culture were collected aseptically. Patient characteristics were presented in frequency tables. Antimicrobial sensitivity patterns were plotted in tables against the bacterial isolates cultured. Chi-square/Fisher's exact test was used to determine any association between patient characteristics, bacterial growth, and antimicrobial sensitivity.

**RESULTS:**

The majority (n = 66; 60%) were males. The median age was 6.3 years (standard deviation, 3.7). The majority of patients 71 (64.5%) had hematologic malignancies, the most common being AML. There was a significant association between severity of neutropenia and hematologic malignancies (*P* = .028). In total, 31/110 (28.2%) blood cultures were positive for bacterial growth. Gram-positive bacteria were more frequent (n = 20; 58.1%). The most common organism was *Escherichia coli* (n = 6; 18.2%), followed by *Staphylococcus aureus* (n = 5; 15.2%). All the isolates were sensitive to linezolid and vancomycin and also showed good sensitivity toward meropenem (n = 10/11; 90.9%). High resistance to cephalosporins was noted with ceftriaxone (n = 5/6; 83.3%), cefepime (n = 4/7; 57.1%), and ceftazidime (n = 3/4; 75%).

**CONCLUSION:**

The most common malignancy associated with febrile neutropenia was AML. Gram-positive bacteria were the most common isolates. There was high resistance to cephalosporins.

## INTRODUCTION

Each year, an estimated 400,000 children and adolescents (age 0-19 years) develop cancer. Higher cure rates are observed in high-income countries, compared with many low- and middle-income countries (LMICs).^1^

**CONTEXT**

**Key Objective**
Are multiple drug-resistant organisms increasingly challenging the management of children with febrile neutropenia on chemotherapy in low-income countries? Management of fever and neutropenia continues to be a challenge in low-income countries. It increases the financial burden on patients because of longer hospital stays and expensive antibiotics. It is also associated with increased mortality due to bacteremia, which proves difficult to treat because of increasing resistance to locally available antibiotics.
**Knowledge Generated**
In this setup, most of the organisms that grew were noted to be resistant to multiple drugs. Furthermore, there was resistance to most cephalosporins, which are the first line of antimicrobial therapy. The overuse/misuse of antibiotics in treating various infections and the lack of routine blood cultures to confirm the presence of bacteremia are some of the contributing factors to the high resistance.
**Relevance**
The findings from this study will guide the development of an antibiogram for patients with febrile neutropenia.

Some of the reasons for lower survival rates in LMICs include delay in diagnosis, inaccurate diagnosis, inaccessible therapy, abandonment of treatment, relapse, death from toxicity, and complications from treatment.^2^ Most childhood cancers are treated with chemotherapy, in addition to surgery and radiotherapy.^3^ Treatment with chemotherapy leads to bone marrow suppression, which is the source of many of the adverse side effects of cancer treatment, including infection and sepsis.^4^ Bone marrow suppression because of chemotherapy leads to disruption in the production of blood cells including WBCs, which form an important part of cell-mediated immunity, hence predisposing patients to infections by bacteria because of a blunted immune response.

Febrile neutropenia is a serious and often fatal complication of chemotherapy and continues to be a significant cause of death among children with cancer. Patients with febrile neutropenia therefore should be investigated early for bacteremia and started on treatment early to avoid and prevent complications.

Children on treatment for cancer who develop chemotherapy-induced neutropenia are often at risk of developing febrile neutropenia as a complication of treatment. In patients who develop febrile neutropenia, the prevalence of bloodstream infections is about 11%-38%, with overall mortality reaching about 40%.^5^ According to the CDC, a bloodstream infection is defined as a laboratory-confirmed blood infection that is not secondary to an infection at another body site.

The challenge in the management of febrile neutropenia is made even more difficult with the increase in antimicrobial-resistant bacterial species. The extensive emergence of multidrug (MDR)-resistant bacteria has increased the burden of morbidity and mortality among cancer patients with bloodstream infections.^6^ The development of antimicrobial resistance is driven by a lack of proper antimicrobial surveillance and stewardship measures.

This study aimed to identify the clinical-demographic characteristics of children with febrile neutropenia admitted with cancer at the Moi Teaching and Referral Hospital (MTRH), the associated bacterial organisms, and their sensitivity patterns.

## MATERIALS AND METHODS

This was a descriptive prospective cross-sectional study that was carried out between June 2021 and April 2022 in the pediatric oncology ward at the MTRH in Uasin Gishu County, Kenya. MTRH is the second largest tertiary hospital in Kenya. It serves a population of about 24 million spread across at least 22 counties in Kenya and also includes patients from parts of Eastern Uganda and Southern Sudan.

The pediatric oncology unit at MTRH has two pediatricians, one medical officer, three registered clinical officers, and general nurses. On average, about 1,000 patients are admitted at the oncology ward per year.

Consecutive sampling was adopted to recruit the participants whose clinical and demographic characteristics were extracted from their medical records. The samples for blood culture were then collected from a peripheral venipuncture site. None of the patients had central lines or peripherally inserted central catheter lines, since these are not used in our setup. About 2-5 mLs of blood was drawn from the patients for blood culture into the BACTEC Peds Plus blood culture collection bottle.

The blood culture samples were incubated in the BACT/ALERT automated blood culture machine. Samples positive for growth were inoculated on three media, which are blood agar, chocolate agar, and MacConkey agar, and then incubated for 18-24 hours, after which the bacterial colonies from the plates were examined and Gram staining was done to identify gram-positive and gram-negative bacterial colonies. These colonies are then directly inoculated into the VITEK 2 COMPACT machine, which uses specific antibiotic susceptibility testing and identification cards to identify the isolates at the species level.

The data collected were analyzed descriptively, and categorical variables were recorded as frequencies and percentages and plotted in tables to show the distribution. A univariate analysis was used to test for association between the outcome variable, which was the blood culture results, and the patients' clinical and demographic characteristics, and to also test for association between severity of neutropenia and categorical variables. A *P* value of <.05 in all analyses done was considered to be statistically significant.

Ethical approval to conduct this study was sought and obtained from the Institutional Research Ethics Committee (IREC) of MTRH-Moi University; Approval No. 0003587. Consent was sought from the patients' parents or legal guardians and assent from subjects who were older than 12 years.

## RESULTS

A total of 112 patients with fever and neutropenia who were admitted to the pediatric oncology ward in Shoe 4 Africa, MTRH, were identified, and 110 were recruited into the study. Two were excluded because we were not able to obtain consent from them. Of the 110 participants who were recruited into the study, about 60% were male while 40% (n = 55) were female. The age of patients ranged from 6 months to 15 years, with a mean of 6.3 (standard deviation, 3.7) years (Table 1). Seventy-one (64.5%) participants had hematologic malignancies.

**TABLE 1:** Patient Characteristics

Characteristic	n = 110, No. (%)
Sex	
Male	66 (60)
Female	55 (40)
Age category, years	
0-4	26 (23.63)
5-10	58 (52.7)
11-15	26 (23.63)
Malignancy type	
Hematologic malignancies	71 (64.5)
Solid tumors	39 (35.5)
ANC, cells/µL	
<500	85 (77.27)
>500 to <1,000	25 (22.73)
Weight-height Z-score	
Normal (z-score >–2)	105 (95.45)
Moderate malnutrition (–3 > z-score <–2)	3 (2.72)
Severe malnutrition (z-score <–3)	2 (1.81)

Abbreviation: ANC, absolute neutrophil count.

The most frequent cancer among the study participants was AML (24.54%), followed by nephroblastoma (22.7%), ALL (21.81%), and Burkitt lymphoma (15.4%; Table 2).

**TABLE 2:** Distribution of Malignancy Type

Variable	Category	Frequency (%)
Diagnosis	AML	27 (24.5)
	Nephroblastoma	25 (22.7)
	ALL	24 (21.8)
	Burkitt lymphoma	19 (17.3)
	Retinoblastoma	3 (2.7)
	Rhabdomyosarcoma	3 (2.7)
	Nasopharyngeal carcinoma	2 (1.8)
	Germ cell tumors	2 (1.8)
	Osteosarcoma	2 (1.8)
	Ewing sarcoma	1 (0.9)
	Hodgkin lymphoma	1 (0.9)
	Neuroblastoma	1 (0.9)

### Factors Associated With Neutropenia

The majority of the patients had severe neutropenia (77.27%) with absolute neutrophil count of <500 cells/μL, and there was a significant association between severity of neutropenia and hematologic malignancies (*P* = .015) when compared with solid tumors (Table 3).

**TABLE 3:** Level of ANC in Relation to Patient Characteristics

Variable	ANC, No. (%)		*P*
	Nonsevere	Severe	
Sex			.642^a^
Male	14 (21.1)	52 (78.8)	
Female	11 (25)	33 (75)	
Diagnosis			.015^a^
Solid	14 (35.9)	25 (64.1)	
Hematologic	11 (15.5)	60 (84.5)	
Diagnosis			.028^a^
AML	1 (4.3)	22 (95.7)	
ALL	6 (26.1)	17 (73.9)	
Other hematologic	4 (16)	21 (84)	
Solid tumors	14 (35)	25 (64.1)	
Age, years, mean (SD)	5.2 (3.3)	6.7 (3.8)	.091^b^
W-H z score			.586^c^
Normal	25 (23.8)	80 (76.2)	
Malnourished	0	5 (100)	

Abbreviations: ANC, absolute neutrophil count; SD, standard deviation; W-H, weight-height.

^a^ Chi square.

^b^ *t* test.

^c^ Fisher's exact.

Severe neutropenia was noted to be more in those with AML (97.5%) in comparison with other malignancies. This was noted to be statistically significant (*P* = .028).

### Bacterial Isolates

Of the 110 blood cultures taken, 31 (28.18%) were positive for bacterial growth. Two of the cultures were polymicrobial, one grew *S. aureus* and *S. epidermidis* and the other grew *S. aureus* and *P. aeruginosa*. The rest were caused by a single bacterial isolate. A total of 33 microorganisms were grown from all the positive blood cultures.

Of the 33 isolates, 57.6% (n = 20) of the microorganisms were gram-positive, while the rest were gram-negative. The most prevalent gram-negative organisms were *Escherichia coli* (n = 6; 18.18%), followed by *Klebsiella pneumoniae* (n = 3; 9.1%). *Staphylococcus aureus* (n = 5; 15.15%) was the most frequent for gram-positive organisms, followed by *Enterococcus faecium* (n = 4; 12.1%; Table 4).

**TABLE 4:** Bacterial Isolates in Relation to Patient Diagnosis

Bacterial Isolates	AML (19.35%)	Nephroblastoma (9.6%)	ALL (25.8%)	BL (19.35%)	RMS (6.5%)	Nasopharyngeal Carcinoma (6.5%)	HL (3.2%)	Osteosarcoma (6.5%)	RB (3.2%)
Gram-positive, No. (%)									
*Staphylococcus aureus* (5)	1 (14.3)		1 (12.5)	1 (14.3)		1 (50)			1 (100)
*Enterococcus faecium* (4)		1 (33.3)		1 (14.3)	1 (50)		1 (100)		
*Staphylococcus hominis* (3)			1 (12.5)	1 (14.3)	1 (50)				
*Staphylococcus haemolyticus* (3)	1 (14.3)	1 (33.3)	1 (12.5)						
*Staphylococcus epidermidis* (3)	1 (14.3)	1 (33.3)	1 (14.3)						
*Streptococcus parasanguinis* (1)	1 (12.5)								
*Enterococcus gallinarum* (1)	1 (14.3)								
Gram-negative, No. (%)									
*Escherichia coli* (6)	3 (42.9)		1 (12.5)	1 (14.3)				1 (50)	
*Klebsiella pneumoniae* (3)			1 (12.5)	2 (28.6)					
*Pseudomonas aeruginosa* (1)					1 (50)				
*Pseudomonas stutzeri* (1)			1 (12.5)						
*Acinetobacter lwoffii* (1)								1 (50)	

Abbreviations: BL, Burkitt lymphoma; HL, Hodgkin lymphoma; RB, retinoblastoma; RMS, rhabdomyosarcoma.

The highest rate of bloodstream infection (BSI) were noted to occur more in those with ALL, AML, and Burkitt lymphoma (25.8%, 19.35%, and 19.35%, respectively) compared with those isolated among nephroblastoma (9.7%) from the total number of positive cultures (n = 31).

Eight (34.8%) isolates were obtained from the total specimen (n = 24) taken from patients with ALL (Table 4).

From Table 4, there is no specific diagnosis that we could point to be associated with a specific organism.

### Antimicrobial Sensitivity Patterns

All the cultured bacterial isolates were found to be resistant to benzylpenicillin and ampicillin at 100%. High resistance rates were also observed toward most cephalosporins, with ceftriaxone at 83.3% and ceftazidime at 75%. However, all gram-positive bacterial isolates were sensitive to both linezolid and vancomycin (Table 5).

**TABLE 5:** Antimicrobial Sensitivity Patterns

Diagnosis	Sensitive, No (%)	Resistant, No (%)	Total, No.
Ampicillin	0	12 (100)	12
Benzylpenicillin	0	17 (100)	17
Gentamicin	10 (35.7)	18 (64.3)	28
Levofloxacin	5 (26.3)	14 (73.7)	19
Linezolid	15 (100)	0	15
Vancomycin	18 (100)	0	18
Amikacin	7 (63.6)	4 (36.4)	11
Amoxiclav	1 (12.5)	7 (87.5)	8
Nitrofurantoin	16 (76.2)	5 (23.8)	21
Rifampicin	9 (75)	3 (25)	12
Clindamycin	6 (54.6)	5 (45.4)	11
Meropenem	10 (90.9)	0	10
Cefepime	3 (42.9)	4 (57.1)	7
Ceftriaxone	1 (16.7)	5 (83.3)	6
Ceftazidime	1 (25)	3 (75)	4
TMP/SMX	1 (12.5)	7 (87.5)	8
Piperacillin/tazobactam	2 (40)	3 (60)	5

Abbreviation: TMP/SMX, trimethoprim-sulfamethoxazole.

## DISCUSSION

This study set out to describe the clinical and demographic characteristics of children with febrile neutropenia on treatment for cancer at MTRH and to identify the common bacterial isolates associated with it. To our knowledge, this is the first study on bacterial isolates in children on treatment for cancer with febrile neutropenia in MTRH.

Male subjects were the majority of patients in this study. These findings were similar to studies done in Egypt, which showed a male predominance (51.3%), and a study done in Indonesia, where there were more male participants (58%).^7,8^

This study noted that the most common malignancies among the enrolled participants were leukemias (AML and ALL), nephroblastoma, and Burkitt lymphoma. This was similar to a study done in India, where the most common malignancies were ALL, non-Hodgkin lymphoma, nephroblastoma, and AML.^9^ Furthermore, it was noted that the most common malignancy type among those with febrile neutropenia in this study was AML. Hematologic malignancies, especially AML, require more intensive myeloablative chemotherapy regimens that are associated with severe myelosuppression, leading to a disruption in the normal hematopoiesis.^10^

From this study, the positive bacterial growth from the blood cultures collected was 28.18%. This was comparable with that of studies done in Colombia,^11^ where the cumulative incidence of BSI was 29.23% (92/315), and a study done in India, which documented a bacterial growth rate of 27.8% from 155 blood culture samples collected.^12^ Another study also supported this, stating that overall bacteremia can be detected in about 20% of patients with febrile neutropenia.^13^ This could be because not all episodes of febrile neutropenia result from bacterial infection and in some cases in the absence of a clinical or microbiological evidence of infection, febrile neutropenia (FN) is marked as a fever of unknown origin.

Gram-positive bacteria were isolated more frequently than gram-negative bacteria in this study. This was also the case seen in different studies done in Sweden, Qatar, and South Africa,^14-16^ which reported a predominance of gram-positive bacteremia in their studies. It can be speculated that over time, the increase in the use of efficient antimicrobial prophylaxis with agents such as fluoroquinolones targeting gram-negative bacteria has led to the emergence of gram-positive bacteria as the dominant species associated with bacteremia in patients with febrile neutropenia. Furthermore, *Streptococci* and *Coagulase-negative Staphylococci* (CONS) reside in the mucosal barriers; therefore, chemotherapy-induced mucositis is associated with early-onset gram-positive bacteremia.^17^

The most common gram-positive organisms seen were *Staphylococcus aureus* (15.15%) and *Enterococcus faecium,* while the most common gram-negative bacteria were *E. coli* (18.18%) and *K. pneumoniae*. This has been documented in other studies done in Italy and India.^10,18^ These findings were also supported by a meta-analysis that reported findings from 17 different studies worldwide, which showed that *E. coli* was the dominant pathogen, constituting a median of 21% of all BSI strains in the 17 studies, followed by *Klebsiella pneumoniae* with a median of 11%, while the common gram-positive species were *S. aureus,* varying from 1% to 13%, and CONS, ranging from 2% to 42%.^19^

Most of the gram-negative organisms had resistance to broad-spectrum cephalosporins, which are usually the first-line treatment for patients with febrile neutropenia in our setup. *E. coli*, *P. aeruginosa*, and *P. stutzeri* were noted to be extended-spectrum beta-lactamase gram-negative bacteria. The increasing resistance to cephalosporins seen in this study was also observed in a study in Lebanon in which 29.3% of the total bloodstream infections were caused by third-generation cephalosporin-resistant gram-negative bacteria.^20^ The reason could be their overuse as broad-spectrum antibiotic coverage for infections.

Additionally, another study stated that many centers no longer considered the use of ceftazidime, a third-generation cephalosporin, as a suitable monotherapy in patients with FN because of its low activity against many gram-positive microorganisms such as *Streptococci*.^21^

All the gram-positive bacteria cultured were sensitive to vancomycin and linezolid, while the gram-negative bacteria were sensitive toward meropenem. This was also observed in studies done in Uganda and South Africa.^16,22^ This is probably because of the fact that they are usually reserved for second-line use and are mostly indicated in cases of severe infections pending blood culture results.

There was a lack of standardization of discs used in the antibiotic sensitivity test. Different antibiotic discs were tested for different isolates.

In some instances, there would be a delay in the incubation of the collected blood samples from the time of collection in the oncology ward; however, they were all stored in a thermostable specimen collection box and kept at room temperature of <30°C after collection.

In conclusion, most of the study participants were between age 5 and 10 years, and the most common malignancy among those with febrile neutropenia was AML. Hematologic malignancies, especially AML, were noted to be associated with febrile neutropenia and BSI gram-positive isolates were slightly more common than gram-negative ones. The most common organisms were *E. coli*, *S. aureus*, and *E. faecium*. All gram-positive organisms were sensitive to linezolid and vancomycin. Gram-negative bacteria had high sensitivity toward meropenem but showed 100% resistance toward benzylpenicillin and ampicillin.

We recommend that empirical antimicrobial management of febrile neutropenia at MTRH Pediatric Oncology ward should consist of meropenem as monotherapy or in combination with an aminoglycoside; linezolid or vancomycin should be reserved for patients with methicillin-resistant *Staphylococcus aureus* (MRSA) or CONS.
